# A Qualitative Analysis of Career Advice Given to Women Leaders in an Academic Medical Center

**DOI:** 10.1001/jamanetworkopen.2020.11292

**Published:** 2020-07-22

**Authors:** Gianrico Farrugia, Christina K. Zorn, Amy W. Williams, Kate K. Ledger

**Affiliations:** 1 Mayo Clinic, Rochester, Minnesota

## Abstract

**Importance:**

Women in academic medicine continue to face systemic obstacles on their paths to leadership. In addition to improving recruitment and advancement opportunities, academic medical centers must facilitate a cultural shift that ensures sustained leadership pathways for women.

**Objective:**

To better understand, from the perspective of women leaders, the workplace and cultural changes that need to take place in academic medicine to increase inclusivity and gender equity.

**Design, Setting, and Participants:**

This qualitative study of 40 women physicians and administrators with senior leadership roles at Mayo Clinic, a nonprofit academic medical center and research institution with campuses in Arizona, Florida, and Minnesota, examined participants’ responses to a question regarding their paths to leadership. Replies were submitted between November and December 2018.

**Main Outcomes and Measures:**

Women were asked to describe career advice (positive or negative) they had received that was the hardest to accept but, in retrospect, turned out to be valuable.

**Results:**

Of 40 participants, 25 (63%) were physicians and 15 (37%) were administrators at Mayo Clinic; 27 (68%) had achieved the role of chair or the administrative equivalent. Career experience ranged from 6 to 40 years. Of the 40 women leaders queried, 38 (95%) provided written responses, which were separated into the 4 following categories: leadership styles are perceived as having gendered qualities, attaining leadership skills involves a strategic learning process, collisions between personal life and the workplace should not deter individuals from pursuing leadership roles, and leadership pathways for women involved hurdles. These categories represented a roadmap illuminating perceptions about the academic medical workplace.

**Conclusions and Relevance:**

These findings link generalizable principles to help to drive new strategies for gender parity. Shifting the culture of academic medicine begins with fully understanding impediments to women’s advancement. The advice women leaders recounted offered a roadmap as well as a glimpse of the extra effort required for women to succeed amid some of the system’s limitations and obstacles. A more complete understanding of gender biases may help to shape future programs to expand inclusivity and establish sustained leadership paths for women.

## Key Points

**Question:**

What advice regarding the workplace and management strategies are perceived as useful to women leaders in academic medicine?

**Findings:**

In this qualitative study based on responses from 40 women leaders at the Mayo Clinic, we found that advice could be grouped into 4 categories: leadership styles are perceived as having gendered qualities, a strategic process is required to gain leadership skills, conflicts between personal life and the workplace will occur and should not be a deterrent, and leadership pathways for women involve inevitable hurdles.

**Meaning:**

The findings of this qualitative study suggest that a long-term view of attaining gender equity for women leaders at academic medical centers will require a combination of programs, including disruptive institutional initiatives and grassroots efforts; 1 approach involves highlighting and disseminating career advice received by female leaders, particularly advice that others have found helpful.

## Teaser

This qualitative study solicits the perspectives of women leaders on the workplace and cultural changes that need to take place in academic medicine to increase inclusivity and gender equity.

## Introduction

In 2017, the Association of American Medical Colleges reported historic admissions numbers at medical schools across the country.^1^ For the first time, more than half (50.7%) of matriculants were women. Similar numbers were repeated the following year, a trend that may reflect increased support for undergraduate women studying science, technology, engineering, and medicine and more encouragement for those who want to pursue medical and research careers.

However, a glimpse at the other end of the career pipeline is still concerning. Women remain underrepresented in positions of leadership at US academic medical centers.^2,3^ In 2018, only 18% of permanent department chairs and 16% of deans were women.^4,5^ In fact, during the last 30 years, the number of women professors in medicine has only grown by 7% (to only 17% of the total).^6,7^

Women in academic medicine continue to face significant systemic inequity. They do not receive promotions or advance to leadership roles at the same rates as men.^8,9^ Gender disparities persist when women physician leaders submit manuscripts for publication and compete for major grants.^10^ Women receive lower salary offers and less institutional research funding and continue to earn less than their male peers with comparable roles and productivity.^11,12^ While work-life balance is a pervasive issue throughout medicine,^11^ women physicians face more stigma than their male counterparts when balancing work with nonwork responsibilities.^13,14^ Furthermore, many report experiencing sexual harassment, hostility, and discrimination at work.^15^

While workplace inequity and inequality affect industries worldwide, they are of particular concern in health care, where they have the potential to affect patient care and health outcomes through stereotypes that diminish the authority of women clinicians (eg, the perception that they care instead of cure).^16^ Gender inequity also contributes to career dissatisfaction and burnout among physicians.^17^ Intersectional analyses show inequalities are further pronounced among women of color.^18^

During the last decade, various strategies have aimed to improve the representation of women leaders in academic medicine. Institutional efforts, referred to as top-down programs, have targeted specific areas, such as compensation inequities or family leave options,^19,20^ while new curricula, such as career development programs, have aimed to improve the retention of women faculty.^21^ Grassroots, or bottom-up, programs have gained traction, providing women with opportunities to broaden skills, pair with mentors, and improve networking opportunities.^7^ For example, Executive Leadership for Academic Medicine has shown improvements in gender inequity.^7^ But top-down efforts can be piecemeal, while grassroots programs require women to invest energy, effort, and personal time, limiting their resources for other priorities.

Overall, individual programs may improve aspects of an unequal system without creating a long-term, sustained path for women to rise to leadership. In fact, barriers to leadership tend to be subtle and embedded throughout women’s careers, from unequal networking opportunities^7^ to the neglect of women’s formal titles when introduced at conferences.^22,23,24^ Young women have few women role models who serve as deans and department or division chairs and have more difficulty than men finding mentors and sponsors.^25,26,27^ Significantly, fewer women than men are involved in hiring committees, which may also affect women’s representation in senior-level positions.^7^

Despite these difficulties, some women physicians do ascend to leadership roles. Along their career paths, many have received advice regarding career advancement and fulfillment. We hypothesized that women leaders would, with the benefit of hindsight, have a different perspective regarding the advice that would be most useful for increasing women’s leadership in academic medicine. Significantly, we were interested in advice that was hard to accept, distinct from generic guidance, such as “be your best self” or “think positively.” A thorough examination of implicit and entrenched perceptions of the workplace may be useful to drive innovative strategies that address inequality.

## Methods

We queried 40 women leaders at Mayo Clinic about their paths to leadership roles. Specifically we asked them about career advice (positive or negative) they had received that was hardest to accept but, in retrospect, turned out to be helpful. Participants were chosen based on their gender and leadership positions. Their responses were submitted by email between November and December 2018; the responses were then completely deidentified for data analysis. The study underwent institutional review board review at Mayo Clinic and was determined to be exempt from the requirement for approval. The data were deidentified and informed consent from respondents was deemed not required by the institutional review board. This qualitative study used an exploratory-descriptive approach and is reported according to the Standards for Reporting Qualitative Research (SRQR) reporting guideline.

### Statistical Analysis

The respondents’ comments were reviewed by the 4 authors and placed in the 4 groups by consensus. No software was used, and no prespecified level of statistical significance was set.

## Results

Of 40 participants, 25 (63%) were physicians and 15 (37%) were administrators at Mayo Clinic; 27 (68%) had achieved the role of chair or the administrative equivalent. Career experience ranged from 6 to 40 years. Of the 40 women leaders queried, 38 (95%) provided a written response, which we binned into 4 categories that represented a roadmap for leadership advancement, illuminating perceptions about the academic medical workplace and how women leaders enter and fit into the hierarchy of authority. The bulleted excerpts below are unedited quotes taken from individual responses. All responses are represented; we chose 1 representative quote when there was significant overlap.

### Leadership Styles Are Perceived as Having Gendered Qualities That May or May Not Resonate With Individuals

A recurrent theme was that leadership styles continue to be perceived as either masculine or feminine according to certain attributes (eg, tough, soft). Perceptions about what might be preferable or effective varied, but respondents often echoed the sense that leadership has distinct, gendered approaches.

- “When I started at Mayo some 40 years ago, I was dismayed with the ‘tough’ persona of many women physicians and administrators. I vowed, if ever a leader, to be an effective, feminine leader. Decades later, when coming into this role, a mentor diplomatically coached me to be a ‘tougher’ leader. After getting over myself and carefully examining her critical feedback, I knew she was right. I relied on my soft skills too much.”
- “As time passed, I gradually learned the value and art of deep listening. It wasn’t enough to listen like my male counterpart listened. Male colleagues share a relatively common language and past experience, so listening between them is not as difficult … the gap between them is relatively narrow. When the gap is bigger due to diversity of past gender-specific experiences, etc, it requires deeper listening to achieve understanding. The observer was right that I was not managing the communication gap that truly existed. I could not accept the approach used by the chair before me. I had to invest in developing new skills, and I am still working on it.”
- “It’s important in leadership positions to make clear and definitive decisions and avoid apologizing for decisions made. This does not mean one can’t be wrong, or that things can’t be renegotiated, but be definitive. I do think women can be apologetic in their communication style, and it takes a long time to get rid of this behavior.”
- “One of the hardest pieces of advice to accept for me was when I was told, ‘Take the credit.’ As women, that is hard for us to do, and we feel we need to be humble and make sure others are promoted. I know that most of my career I haven’t felt that I actually deserved the credit for successes to which I have contributed or have been responsible for. I have learned, though, there is a gracious way to ‘take the credit’ that doesn’t diminish me or my colleagues.”

### Attaining Leadership Skills Involves a Strategic Learning Process With Decision Points

Respondents suggested that they viewed their own behaviors as modifiable to be successful. The advice they received prompted them to gain new perspectives, and they saw themselves as being capable of making personal changes at an appropriate moment to promote their achievement or increase their effectiveness, even when the changes were difficult to make.

- “‘Be empowered and control your position.’ This relates more to feeling intimidated. In my specific experiences, it was scenarios with respected male leaders that had a lot of power. This advice was given to me early on (before gender equity was openly discussed), and it took time to conquer. Feeling confident and contributing will build respect and trust with all leaders in the long run. Offering a different opinion or perspective (when appropriate) has also proven to be helpful in my past/current experiences. This is not always an easy thing to do, especially early on in a career.”
- “Good advice I received but did not immediately follow was—you need to fact-check the stories you’re telling yourself. I had no idea all the stories in my head. One story I told myself is that high prep anxiety will equal success, which is wrong. Another is people don’t see me as a leader—if I go for this position, people will think I’m full of myself. It goes on and on. We need to find our trusted truth-tellers.”
- “Avoid ‘I think’/‘I believe’/‘I feel’ or other apologetic or negotiable phrases like ‘on the other hand’/‘however’ or ‘but’/‘another way of looking at things.’”
- “A mentor told me I needed to be more assertive and better at ‘tooting my own horn.’ At the time, I did not agree with him, as culturally my style is to be understated.… However, a while back a female leader told me that my reputation was that I am good at identifying problems but not necessarily good at finding solutions. I stewed on this long and hard, and the question I had to ask myself was, why does this ‘misperception’ exist? What am I doing (or not doing) that has led to this perception? This led me to reflect on the advice.… Working behind the scenes is fine, but in a competitive world you have to be assertive and to claim ownership, as others will not toot your horn for you.”
- “If I don’t know the answer, I need to own it on the spot and either help redirect to the person who will know or do my research and follow up promptly once I have the answer. I have found that this builds respect, and in the end, honesty and transparency will go further.”
- “A mentor told me, ‘It is always nice to be asked, but if you fill up all of your time by saying yes to others, you’ll rarely have room for what’s important to you.’ Her advice helped to change my brand from someone who is ‘helpful’ to someone ‘who knows where she wants to go.’”
- “The best ‘hard’ advice I received was, ‘You need to stop sprinting. Mayo Clinic is like a marathon.… [Y]ou need to slow down, be more thoughtful and strategic, or you will burn yourself and others out.’”

### An Individual’s Personal Life Will Collide With the Workplace and the Conflict Should Not Be a Deterrent to Pursuing a Leadership Role

As respondents recounted effective advice, they revealed a workplace in which the demands of leadership conflict with an individual’s personal life. Women described choosing to assert themselves, either to fulfill a personal need or to subscribe to the workplace, or artfully navigating a balance between both to establish work-life boundaries.

- “There is no ‘right’ time to have a baby. You just MAKE it work out.”
- “What if you gave 90% to 95% instead of 120%? My false belief was my success was because I always made sure—to excess—that my slides and everything else were perfect. So, things were great, but I was getting burned out. My coach said that my 95% was probably the same as most people’s 120%—no one would notice, but it would make a huge difference for me. It was very scary to do—to start working on talks a month ahead of time instead of 3 months ahead, but as it turns out, my talks were better because I was less uptight and overpracticed. I had more time, but still a good presentation.”
- “My hardest-to-accept advice that is spot on is, ‘Stop pursuing perfection.… [I]t is false and often makes things worse.’ This is in the context of having difficulty ‘finishing’ a treatment plan for a patient or a departmental project. I have worked hard to transition to pursuing excellence, not perfection. Excellence in my opinion is not being perfect or the best, but always trying to be better. It is a fine distinction, but can make an enormous difference in effectiveness as a clinician, researcher, and leader as well as wife and mother.”
- “In addition to my personal and regular professional responsibilities, I was helping with a few large projects for colleagues that were not tied to my role directly, and I was mentoring 2 junior colleagues on a weekly basis. I was spending a significant amount of time on other people’s stuff, especially when the requestor was someone more senior than me, when there was flattery involved in the ask, or when I felt an obligation to ‘be nice.’”
- “It’s not that you can’t have it all—you don’t want it all. Figure out what you want and strategically make your own luck.”

### Leadership Pathways for Women Involve Inevitable Hurdles and Require Courage to Follow or Ignore Advice

Many respondents commented about ongoing obstacles on their paths to leadership. Many did not see the advancement of their careers as guaranteed or even supported, despite believing in their own skills and capabilities.

- “I was told/asked to take my hat out of the ring for the following reasons: it would pigeonhole me into a role that would be hard to be promoted from (too much siloed experience); and I’d probably get it if I applied but that I would be bored doing this same role for 20 more years of my career. At the time I thought my leader thought I was too young to take it on; he wanted his protégé in the role and wasn’t willing to go with the higher performance; and I often wondered if being the spouse of an MD made people question my long-term commitment. Instead, I was asked to take a lateral position … (which ultimately led me on a different, but ultimately more successful, path).”
- “For me, my age and gender were what held me back, and I almost did not put my name in the running for the [leadership] position. When the email from the search committee chair came, I was very interested and thrilled, and I felt in my heart that this was the right moment for me and the right next step that I was looking for. However, I immediately thought that I am ‘too young’ for a chair and, definitely too young for a woman chair. Somehow, in my mind, I would not have questioned a younger man who wanted to compete for this, but I did not envision that I could. I am sorry to say this, I am not usually someone who would make a judgment based on gender or age for someone else, but, when it came to me, I did. Furthermore, I tried to seek advice from a senior leader, and they confirmed what I initially thought and told me not to come for an interview. On the other hand, 2 of my close mentors, who know me very well, pushed me to apply and convinced me that the old preconceptions are not what defined me … that my drive, my passion, my ability to connect with people are more important. It was a very difficult decision for me to even consider applying for this position, and I would not have had the confidence to do so if it were not for my mentors. Once I came for the interview, I knew that this is what I want to do and that I can definitely do it, just as well as others could, but I would not have considered it without them encouraging me.”
- “‘Stiffen your backbone.’… It was the best advice I ever got. It told me that I was the leader, I had to lead, and I could not afford to get overwhelmed no matter how difficult the situation. I did just that.… I stiffened my backbone. I felt a new sense of courage and resolve. To this day, I still hear those words in my mind. It reminds me to gird my resolve and get on with things!”

## Discussion

A growing body of literature is revealing the extent of gender inequity in academic medicine and its association with women’s professional advancement. Several recommendations have included institutional changes to promote gender equity and to improve the representation and retention of women in leadership roles.^28^ These include developing plans for recruitment, a better infrastructure for maternity leave and parental leave, and greater efforts to provide salary transparency.

Other efforts have focused on improving women’s opportunities by increasing their exposure to skill building, mentoring, and networking. Some programs aim to create a well-trained and prepared leadership cadre from within, such as a successful pilot program at Mayo Clinic, where we work. The program has identified a group of 20 women and professionals from underrepresented minority groups who are candidates for advancement within the academic ranks. The participants are assigned mentors and given protected time for 12 months to build leadership skills, professional networks, and research and publication portfolios, with the goal of academic promotion.

However, deep-rooted cultural changes will be necessary to retain women in high-level roles. Some academic medical centers are acknowledging the imperative to make the work environment more inclusive and reduce hostility and harassment. The Mayo Clinic became a founding signatory of TIME’S UP Healthcare to express a commitment to advancing equity and inclusion in health care.^29,30^ Through this effort, the Mayo Clinic joined other health care institutions placing a priority on creating a safer, more respectful, and equitable workplace.

This article describes another approach to achieving gender equity in leadership positions, namely, asking women leaders about effective advice they received and then disseminating the results. While a combination of top-down and bottom-up efforts may make changes, academic medical centers must get at the core of the culture that is hard to move. Shifting the culture begins with fully understanding it and considering the wide range of impediments to advancement. We found the advice women leaders recounted offered a glimpse of the extra effort required for women to succeed; it also shed light on some of the system’s limitations. A more complete understanding of gender biases and the often subtle way those biases are manifested^31^ may help to direct and shape future programs to expand inclusivity. For instance, it is important to understand the continuing expectation that effective leadership has gendered qualities. This expectation may force some women to emulate or reject certain behaviors (ie, being tough or soft), regardless of their skills or personal styles. Similarly, women leaders may continue to be judged by those traits, rather than by outcomes. A more complete understanding of gendered expectations and the role they play may prompt new programs to minimize the effects of gendered leadership. Such programs may help cultivate a workplace that accepts and supports a wider range of leadership styles, invites a diversity of opinions and approaches, and even reduces toxic behaviors, such as harassment.

Having greater clarity regarding the obstacles that exist in achieving work-life balance may shape new policies and the development of new top-down programs. These may include innovative opportunities for women physicians who are also caregivers. The Mayo Clinic as well as some other institutions have a pilot program offering grants to clinician-researchers who are also primary caregivers at home, in which funding supports a particular project or offsets clinical hours. Institutions that have implemented policies to support extended maternity leave, lactation sites, remote work, part-time employment, and parental leave, which provides caregiving opportunities for men as well, display adaptability to employees’ work-life balance, changes that have been found to benefit both women and men.^32^ Clearly more can and must be done in this area to help promote sustainable changes, remove stigma, and equalize opportunities.

If we aim to make radical, disruptive, and effective changes in gender inequality, we must continue to examine all aspects of the workplace environment in academic medicine and be honest about the current obstacles. We must also elucidate a better intersectional understanding about how the dynamics of other power hierarchies, such as race, sexuality, or gender identity, affect access to the leadership pipeline. The results will help to determine the best combinations of policies, programs, and approaches to address inequality in academic medicine and drive innovative change. Highlighting and disseminating career advice received by female leaders, particularly those recommendations that turned out to be helpful will inspire and guide strategies to advance women’s leadership in academic medicine. A summary of recommendations is presented in the Box.

Box. **Recommendations to Improve Women’s Leadership Pipeline in Academic Medicine**

- While a combination of institutional and grassroots programs will be necessary to address the wide range of inequity in the workplace, institutions must make a deliberate effort to get at the core of the culture that is hard to move.
- Highlighting and disseminating career advice received by female leaders, particularly those recommendations that turned out to be helpful, is a viable strategy to support women’s rise to leadership.
- Achieving a better understanding of the implicit barriers women face may drive entirely new strategies for achieving equity and ensuring sustained leadership pathways for women.

### Limitations

This study has limitations, including the relatively small size of the cohort, that the data collection was from only 1 institution, and that our focus was on gender without other intersectional dynamics, such as race. Furthermore, this study did not distinguish between the ages of respondents. Gender equity has changed during the last decades, and certain advice might reflect the periods in which it was given.

### Conclusions

In this study, the advice that women leaders received offered a roadmap as well as a glimpse of the extra effort required for women to succeed amid some of the system’s limitations and obstacles. Shifting the culture of academic medicine begins with fully understanding these impediments, which could help to shape future programs that expand inclusivity and establish sustained leadership paths for women.
